# Impacted Mandibular Third Molar Migration in the Presence of Idiopathic Osteosclerosis: A Rare Case Report

**DOI:** 10.3390/dj14020104

**Published:** 2026-02-12

**Authors:** Jure Martinić, Petra Stazić Kunčić, Tanja Gović, Ante Pojatina, Ante Mihovilović, Daniel Jerković

**Affiliations:** 1 Zagreb County Public Health Dental Office, 10000 Zagreb, Croatia; juremartinic98@gmail.com; 2 Department of Oral and Maxillofacial Surgery, Clinical Hospital Centre Split, 21000 Split, Croatia; petra.stazic5@gmail.com (P.S.K.); apojatina@gmail.com (A.P.); amihovilovic@kbsplit.hr (A.M.); 3 Department of Oral Surgery, University of Split School of Medicine, 21000 Split, Croatia

**Keywords:** idiopathic osteosclerosis, impacted third molar, tooth migration, jaw pathology, inferior alveolar nerve, oral surgery, paresthesia

## Abstract

**Background:** Idiopathic osteosclerosis is a non-expansile bone lesion of unknown etiology, mostly detected incidentally on dental radiographs. It is typically asymptomatic and does not require intervention. **Methods:** This case report presents a rare clinical presentation in a 30-year-old female patient in whom enlargement of idiopathic osteosclerosis was associated with the progressive migration of an impacted mandibular third molar into close proximity with the inferior alveolar canal. Consequently, this caused paresthesia of the lower lip and chin and required surgical intervention. **Results:** The diagnosis was confirmed through histopathological examination following surgical removal of the tooth and bone biopsy, which verified the presence of idiopathic osteosclerosis and excluded other possible differential diagnoses. **Conclusions:** The postsurgical period was uneventful, and the patient reported no neurosensory disturbances after surgical treatment.

## 1. Introduction

Tooth impaction represents a frequent developmental disturbance in the eruption process, often posing diagnostic and therapeutic challenges in dental and maxillofacial practice. Its prevalence and potential complications make it an important topic in both clinical dentistry and oral surgery [1,2]. Impacted teeth are common in both adolescents and adults, with mandibular third molars being the most frequently affected, followed by maxillary third molars and canines [1]. A 2016 systematic review reported a global prevalence of 24.4% for impacted third molars in individuals over 17 years of age. More recently, a 2024 meta-analysis estimated the pooled prevalence to be 36.9% per subject and 46.4% per tooth [1,3]. Although often asymptomatic, impacted mandibular third molars are frequently associated with complications such as pericoronitis, periodontal disease, caries, cysts, and tumors [4].

Numerous studies have investigated the etiology of tooth impaction, emphasizing factors such as space deficiency in the dental arch, ectopic eruption pathways, and local pathological conditions. Among the latter, idiopathic osteosclerosis (IO) has been occasionally discussed as a potential contributing factor [3,5,6]. Idiopathic osteosclerosis (IO) is a non-expansile, well-defined radiopaque bone lesion of unknown origin, most often discovered incidentally during routine radiographic examination [6,7]. The lesion is also referred to as a dense bone island, enostosis, bone scar, or focal periapical osteopetrosis, and is considered the intrabony equivalent of exostoses due to its localized overgrowth of cortical bone into the cancellous space [6]. It is commonly found in the mandibular premolar–molar region and may appear as round, elliptical, or irregular in shape, with either homogeneous or mixed radiopacity [6]. Although IO is typically asymptomatic, it can occasionally lead to pathological tooth migration, root resorption, or ectopic eruption due to its localized impact on surrounding dental structures [6,7,8,9]. Among local factors influencing tooth impaction, IO has been occasionally associated with abnormal tooth migration. The increased density and rigidity of the bone within IO lesions may alter normal eruptive forces, potentially pushing an impacted tooth deeper into the jaw. Such changes can complicate eruption pathways and may affect adjacent structures, including the mandibular canal, highlighting the importance of recognizing IO in diagnostic and therapeutic planning [6,10,11,12].

This case report describes a rare clinical presentation of mandibular third molar migration associated with localized IO. The case is further distinguished by the intimate proximity of the impacted tooth to the mandibular canal, resulting in lip and chin paresthesia which presents unique diagnostic and therapeutic considerations.

## 2. Case Report

**Figure 1:**
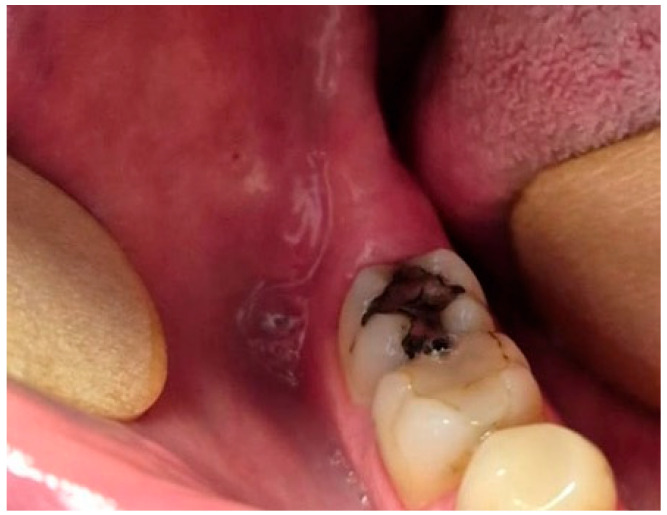
Intraoral view of the posterior right mandibular region showing no visible signs of swelling or inflammation.

Informed consent was obtained from the patient for the use of clinical data and imaging for present case report. A 30-year-old female patient was referred to the Department of Oral Surgery due to a radiographically evident lesion in the posterior right mandibular region and the observed migration of an impacted mandibular third molar. The patient was referred by her general dentist following the onset of numbness in the right lower lip and chin region. She denied pain, swelling, or signs of pericoronitis. The patient’s medical history was noncontributory, and no systemic diseases or previous trauma were reported. Extraoral examination revealed no facial asymmetry, swelling, or tenderness. Preoperative neurosensory function was assessed clinically by comparing tactile and pin-prick sensation of the lower lip and chin bilaterally using a dental probe. Reduced sensation was noted on the right side compared to the contralateral side. Intraorally, the mucosa was intact with no signs of inflammation or infection in the posterior right mandibular region. Upon periodontal probing, a deep periodontal pocket was detected distal to tooth 47, suggesting a possible osseous communication with the underlying impacted tooth (Figure 1). However, she reported paresthesia affecting the lower lip and chin at the time of examination.

**Figure 2:**
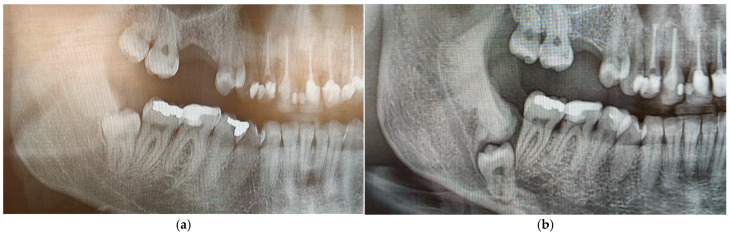
Radiographic comparison of the condition from three years ago (April 2022) (**a**) and the current state (June 2025) (**b**), showing a difference in the depth of impaction of tooth 48 and the size of the bony lesion.

The initial panoramic radiograph was obtained in April 2022 during a routine dental examination, at which time the patient was asymptomatic and reported no neurosensory disturbances. In June 2025, following the onset of lower lip and chin paresthesia that led to referral to an oral surgeon, a follow-up panoramic radiograph and CBCT examination were performed, enabling assessment of radiographic changes over a three-year period. The earlier panoramic image showed an impacted right mandibular third molar (tooth 48) positioned vertically and at a safe distance from the inferior alveolar canal, with a small, well-defined radiopaque area distal to the tooth. In contrast, the current panoramic radiograph revealed an enlargement of this radiopaque zone and a deeper position of the impacted tooth (Figure 2a,b).

**Figure 3:**
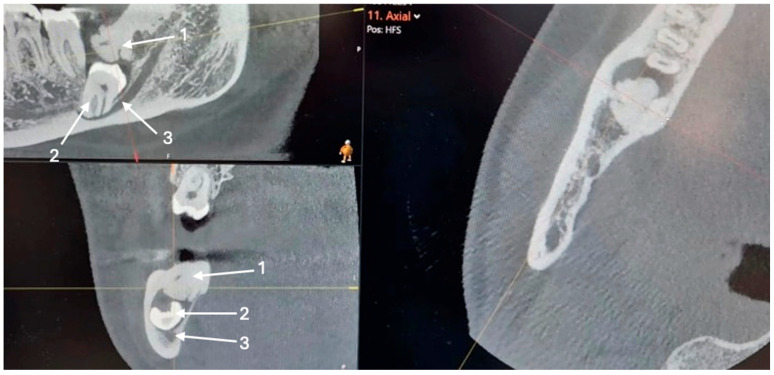
CBCT image showing the impacted tooth 48 and associated bone changes, where 1 indicates a sclerotic bone change, 2 an impacted mandibular third molar (tooth 48), and 3 the inferior alveolar nerve canal.

Cone-beam computed tomography (CBCT) was subsequently performed to assess the extent of the lesion and its relationship to the mandibular canal. The CBCT scan demonstrated a deeply impacted mandibular third molar in direct contact with the superior border of the inferior alveolar canal. A broad osseous communication was observed between the distal root of tooth 47 and the crown of tooth 48, forming a bony pocket. An extensive area of increased bone density was also noted distal to tooth 48, extending toward the ascending ramus of the mandible. The lesion exhibited homogeneous radiopacity comparable to cortical bone, with intact cortical margins and no signs of bone expansion or radiolucent halo, consistent with idiopathic osteosclerosis (Figure 3).

The comparison between the two radiographs confirmed progressive changes over time, with increased bone density and deeper tooth impaction, suggesting that idiopathic osteosclerosis may have contributed to the gradual displacement of the third molar within the mandibular bone.

The radiopaque lesion warranted further investigation, as such sclerotic changes may reflect a range of entities from benign to malignant, especially when associated with lower lip and chin paresthesia [13]. Preoperative assessment included careful evaluation of radiographic features: the lesion was well-defined, non-expansile, and homogeneous in density, with intact cortical borders and no evidence of periosteal reaction or surrounding bone destruction. These imaging characteristics were considered inconsistent with aggressive or malignant conditions such as osteosarcoma or metastatic carcinoma. Clinically, the absence of rapid growth, pain, or systemic symptoms further supported a benign etiology. Nevertheless, due to the lesion’s proximity to the impacted third molar and inferior alveolar nerve, an intraoperative bone biopsy was performed to definitively establish the diagnosis. Considering the lesions proximity to the mandibular canal and deeply impaction, a surgical intervention under general anesthesia was planned. The treatment included surgical removal of the impacted tooth 48 and a bone biopsy of the sclerotic area to establish definitive diagnosis. Preoperative anesthesiologic evaluation was completed, and the procedure was performed uneventfully. Under general anesthesia, a full-thickness mucoperiosteal flap was elevated to expose the surgical site. Bone removal was limited and performed carefully using a conventional rotary instrument under copious irrigation, with particular attention paid to the proximity of the inferior alveolar canal. A small portion of the dense sclerotic bone was removed for histopathological examination. Given the close relationship between the impacted tooth and the inferior alveolar nerve, a coronectomy-assisted extraction approach was employed. Surgical manipulation was restricted to the coronal and pericoronal region, avoiding excessive apical bone removal in the vicinity of the inferior alveolar canal. The crown of the mandibular third molar was sectioned and removed first, while the roots were subsequently luxated and extracted with minimal force to reduce the risk of nerve injury. No direct manipulation of the inferior alveolar nerve was performed. The extraction socket was thoroughly irrigated, hemostasis was achieved, and the flap was repositioned and sutured using resorbable sutures.

The patient received both verbal and written postoperative instructions. Postoperatively, the patient was prescribed amoxicillin–clavulanic acid due to the extent of the surgical procedure. Analgesics were taken as needed for pain control. In addition, a vitamin B-complex supplement (Neurobion P&G Health Austria GmbH & Co. OG, Spittal der Drau, Austria) was administered for a period of four weeks to support neurosensory recovery. Oral hygiene measures included rinsing with a 0.12% chlorhexidine aqueous solution twice daily for 1 min over a period of 7 days.

**Figure 4:**
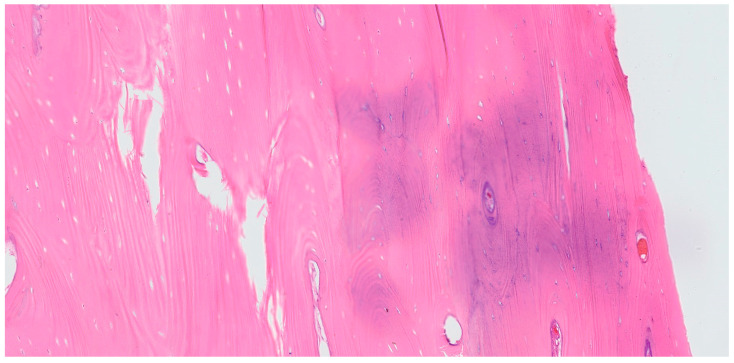
Histopathological section of the sclerotic mandibular lesion showing dense lamellar bone with normal osteocytes, without inflammation or atypia, consistent with idiopathic osteosclerosis (hematoxylin and eosin staining, original magnification ×40).

**Figure 5:**
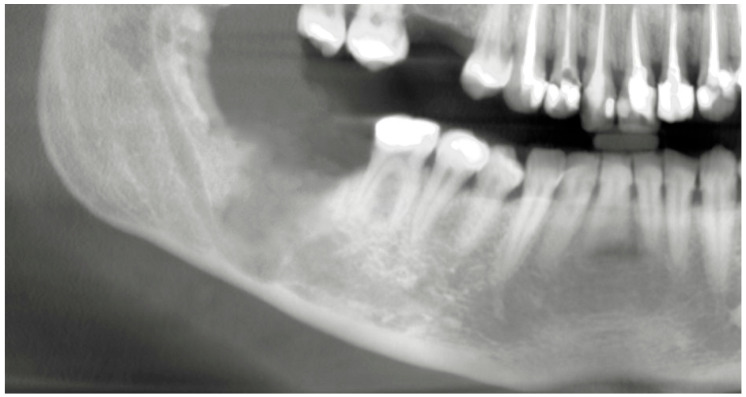
Six-month postoperative panoramic radiograph showing normal bone healing without radiographic evidence of lesion progression.

Histological examination of the bone biopsy confirmed the diagnosis of idiopathic osteosclerosis. The lesion consisted of dense, mature lamellar bone with minimal osteocyte lacunae and absence of osteoblastic or osteoclastic activity. No inflammatory infiltrates, necrosis, or cellular atypia were observed. The bone trabeculae were well-organized and compact, consistent with a benign sclerotic lesion. These features are characteristic of idiopathic osteosclerosis and allowed definitive exclusion of malignant or other pathological entities (Figure 4). At the one-week postoperative review, the patient reported persistent but notably reduced paresthesia in the right lower lip and chin. Wound healing was uneventful, with no signs of infection or swelling. Postoperatively, neurosensory evaluation was performed using the same comparative method at follow-up visits. Progressive improvement was observed, and at the one-month follow-up, symmetric tactile sensation was present bilaterally, indicating complete recovery of sensory function. After two weeks, the paresthesia had almost completely resolved, and at one month postoperatively, normal sensory function was fully restored. At the six-month postoperative clinical and radiographic follow-up, normal bone healing was observed with no signs of lesion progression (Figure 5). The patient was subsequently scheduled for an additional follow-up examination one year later to continue long-term monitoring.

## 3. Discussion

In our case, we present a 30-year-old female patient with IO in the mandibular right posterior region, located above an impacted third molar. The condition had remained asymptomatic for years, until the patient developed paresthesia of the lower lip and chin—a symptom likely related to the close proximity of the impacted molar to the mandibular canal. IO is reported in the literature with a prevalence ranging from 4% to 31%, which largely depends on the diagnostic criteria applied. The variation stems from differences in study designs—some include radiopacities related to occlusal trauma or pulpal inflammation, while others consider only true idiopathic lesions [14]. The mandible, particularly the premolar and molar regions, is the most common site of IO while the etiology and biological behavior of IO remain unclear [7,9,14]. Proposed contributing factors include retained fragments of primary tooth roots, localized bone deposition as a response to abnormal occlusal forces, or anatomical variations similar to tori [7,9].

Serial radiographs, including a panoramic image from several years ago, a recent panoramic, and a CBCT scan, showed clear changes in the position of the impacted tooth and the size of the sclerotic area. Initially, tooth 48 was positioned more superficially and further from the mandibular canal, with only a slight radiopaque area distal to it. On the latest images, both the radiopacity and the tooth’s depth had noticeably increased. While idiopathic osteosclerosis is a non-expansile lesion and does not exert active pressure, its increased bone density and rigidity may alter local bone remodeling dynamics and eruption pathways [7,8,9]. Rather than generating an active displacement force, IO may act as a mechanical barrier, redirecting residual eruptive or eruption-related forces of the impacted tooth along a path of least resistance. Such a passive biomechanical mechanism could result in gradual tooth migration, including movement toward deeper mandibular regions or closer proximity to vital anatomical structures such as the inferior alveolar canal. Similar associations between idiopathic osteosclerosis and abnormal tooth positioning, ectopic eruption, or root resorption have been reported in the literature, supporting the concept that IO may interfere with normal tooth positioning through indirect mechanical and biological mechanisms rather than active expansion. Similar findings were described by Marques-Silva et al. where ectopic tooth eruption associated with idiopathic osteosclerosis caused root resorption of the adjacent tooth [7]. Oshima et al. reported two clinical cases involving long-term follow-up of mandibular idiopathic osteosclerosis in adolescents, where IO was associated with abnormal root development and malposition of the affected teeth [8]. Natarajan et al. presented a rare case involving a transmigrated impacted mandibular first molar associated with IO [9]. All of the above reinforces the idea that IO can have mechanical effects on adjacent structures and, in some instances, interfere with normal tooth positioning.

Previous studies suggest that idiopathic osteosclerosis (IO) is a stable, non-progressive lesion in asymptomatic adults, with no significant morphologic changes observed on CBCT follow-up after 12 months. Nevertheless, our case highlights the importance of long-term radiographic monitoring, as notable alterations in tooth position and lesion relationship to adjacent structures became evident only after several years. Therefore, while IO is generally regarded as a “leave-me-alone” lesion, individualized follow-up strategies should be considered, particularly when IO is associated with impacted teeth or neurovascular proximity [14,15].

In the present case, an intraoperative bone biopsy of the lesion was performed, and histopathological analysis confirmed the diagnosis of IO, thereby ruling out other potentially serious pathologies. There are striking radiological similarities between osteosclerotic lesions and various other radiopaque entities of the maxillofacial bones. These lesions must be carefully differentiated from conditions such as central exostoses, osteomas, mature central ossifying fibromas, cemento-osseous dysplasias, complex odontomas, gigantiform cementomas, osteopoikilosis (spotted bone disease), osteosarcoma, metastatic carcinomas, osteomyelitis, and medullary bone infarction. Careful evaluation of imaging characteristics—including well-defined borders, non-expansile nature, homogeneous radiopacity, and absence of periosteal reaction—helped preliminarily differentiate IO from aggressive or malignant conditions. Nevertheless, definitive diagnosis relied on histopathological confirmation. This distinction is of critical importance, as some of these entities are malignant, and accurate diagnosis is essential to avoid inappropriate or overly aggressive treatment [13,16,17].

Furthermore, sclerotic bone changes may also occur secondary to chronic inflammatory processes, such as condensing osteitis, several clinical, radiological, and histopathological features in the present case argue against an inflammation-induced etiology. Condensing osteitis is typically associated with a non-vital tooth or chronic periapical or periodontal infection and often demonstrates ill-defined radiopacity reflecting a reactive process [18]. In contrast, the affected teeth in this case showed no clinical signs of active infection and no history of pericoronitis was reported. Importantly, histopathological examination revealed dense, mature lamellar bone without inflammatory infiltrates or osteoblastic reaction, which is inconsistent with condensing osteitis. The observed periodontal pocket and bony defect are therefore considered secondary to the progressive migration of the impacted third molar rather than the primary cause of the sclerotic lesion.

Idiopathic osteosclerosis lesions are generally stable over time and often persist for years without causing symptoms, so surgical intervention is usually not required [14]. However, biopsy and treatment may be indicated if the lesion shows progressive enlargement or is associated with clinical symptoms [9]. In our case, we decided on surgical removal of the impacted third molar and bone biopsy because the lesion had increased in size over time and was complicated by the migration of the tooth into close proximity with the mandibular canal, which resulted in paresthesia of the lower lip and chin. In contrary, Natarajan et al. did not perform surgical removal of the transmigrated tooth due to the absence of symptoms and the proximity of the tooth to mandibular inferior border and mandibular nerve, highlighting how clinical decisions may differ depending on individual case characteristics [9].

This case report has several limitations, including its single-case design, the absence of standardized quantitative neurosensory testing, and retrospectively obtained initial radiographic data without a predefined follow-up protocol.

## 4. Conclusions

Although IO is usually of no clinical concern, this case demonstrates that, in rare instances, it may contribute to significant anatomical changes over time. Such cases underline the importance of long-term radiographic monitoring, especially when IO is detected near impacted teeth or vital anatomical structures. Early recognition of progression and timely intervention may help prevent complications and guide appropriate management. Additionally, when biopsy is indicated, histopathological analysis is essential to confirm the diagnosis and exclude other potentially serious conditions.

## Data Availability

The data are not publicly available due to due to patient confidentiality.
